# Supplementary material for: Ral GTPases in Schwann cells promote radial axonal sorting in the peripheral nervous system
Source: J Cell Biol. 2019 Jun 14;218(7):2350–69. doi: 10.1083/jcb.201811150 (PMC6605813; doi:10.1083/jcb.201811150)
Supplement: Supplemental Materials (PDF) [file JCB_201811150_sm.pdf]

Supplemental material

Ommer et al., https://doi.org/10.1083/jcb.201811150

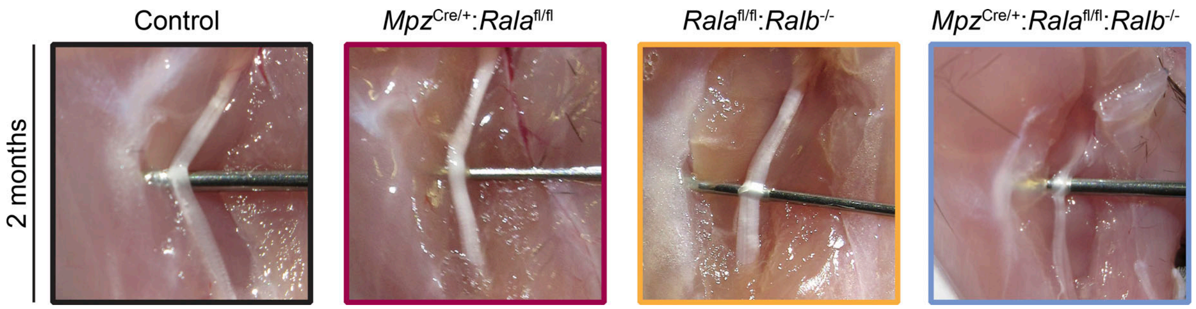

Figure S1. **Sciatic nerves of *Mpz*$^{Cre/+}$:*Rala*$^{fl/fl}$:*Ralb*$^{-/-}$ mice are unusually thin and partially translucent.** Sciatic nerves of control, *Mpz*$^{Cre/+}$:*Rala*$^{fl/fl}$, *Rala*$^{fl/fl}$:*Ralb*$^{-/-}$, and *Mpz*$^{Cre/+}$:*Rala*$^{fl/fl}$:*Ralb*$^{-/-}$ mice at 2 mo of age were exposed to evaluate the general appearance (exemplary pictures). Nerves of *Mpz*$^{Cre/+}$:*Rala*$^{fl/fl}$:*Ralb*$^{-/-}$ mice were thinner and more translucent than those of the other genotypes (consistent observation in ≥30 mice per genotype).

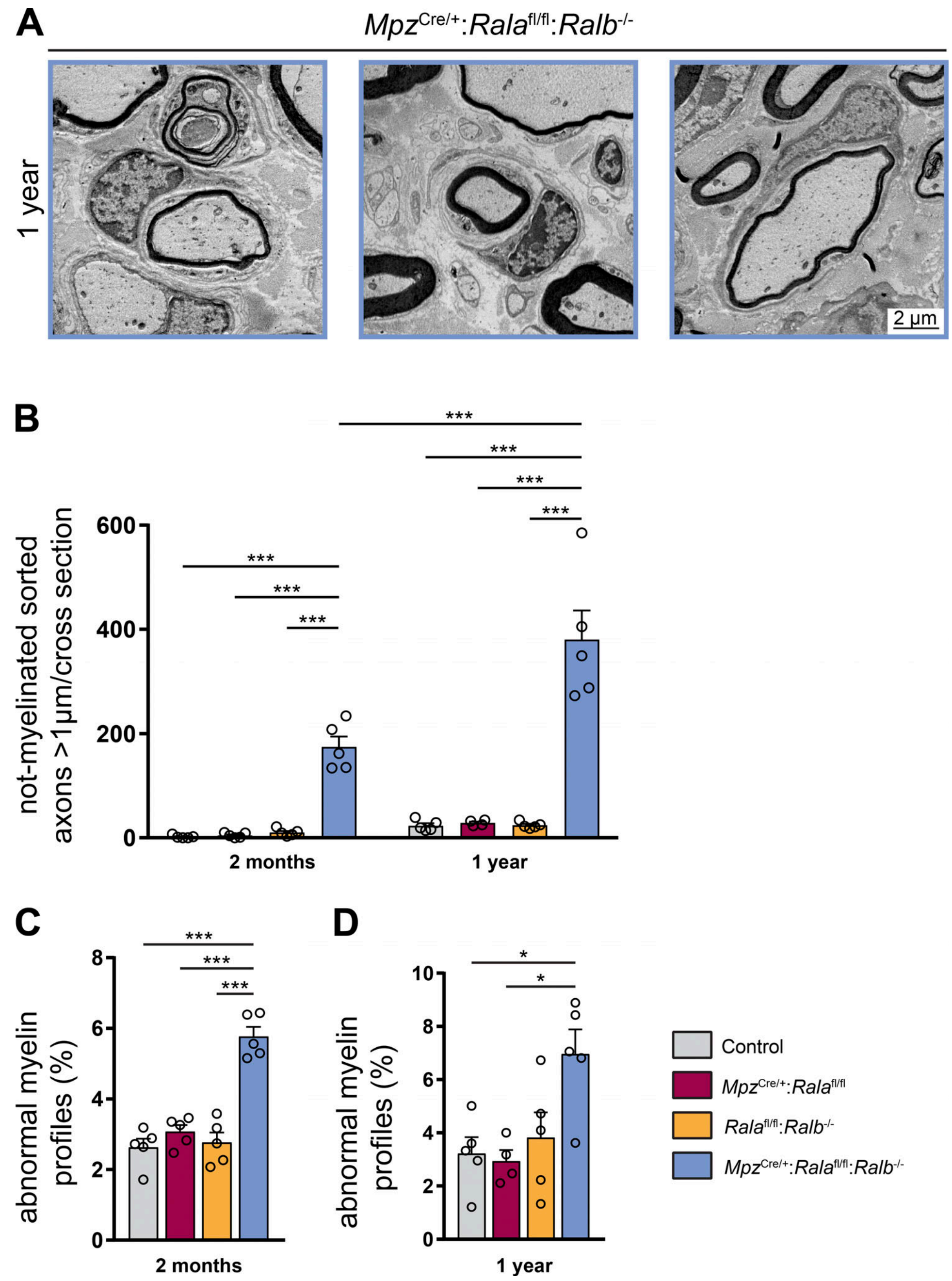

Figure S2. **Evidence for de- and remyelination in sciatic nerves of *Mpz*$^{Cre/+}$:*Rala*$^{fl/fl}$:*Ralb*$^{-/-}$ mice. (A)** Electron micrographs of sciatic nerves of 1-yr-old *Mpz*$^{Cre/+}$:*Rala*$^{fl/fl}$:*Ralb*$^{-/-}$ mice showing thinly myelinated axons and onion bulb–like structures as typical features of attempted remyelination. **(B)** Quantification of the number of not-myelinated sorted axons with a diameter >1 µm per sciatic nerve cross section in 2-mo-old and 1-yr-old animals (dataset of 2-mo-old animals is shown again in Fig. 2 H). n = 4 (only *Mpz*$^{Cre/+}$:*Rala*$^{fl/fl}$ at 1 yr) or 5 mice per genotype, with one complete cross section per animal analyzed. Two-way ANOVA with Tukey's multiple comparisons test. **(C and D)** Quantification of abnormal myelin profiles (infoldings, outfoldings, tomacula, and detached myelin sheaths) expressed as percentage of total number of myelinated axons in 2-mo-old (C) and 1-yr-old (D) animals. n = 4 (only *Mpz*$^{Cre/+}$:*Rala*$^{fl/fl}$ at 1 yr of age) or 5 mice per genotype, with one complete cross section per animal analyzed. One-way ANOVA with Tukey's multiple comparisons test. All data are shown as mean ± SEM. *, $P < 0.05$; ***, $P < 0.001$.

https://doi.org/10.1083/jcb.201811150

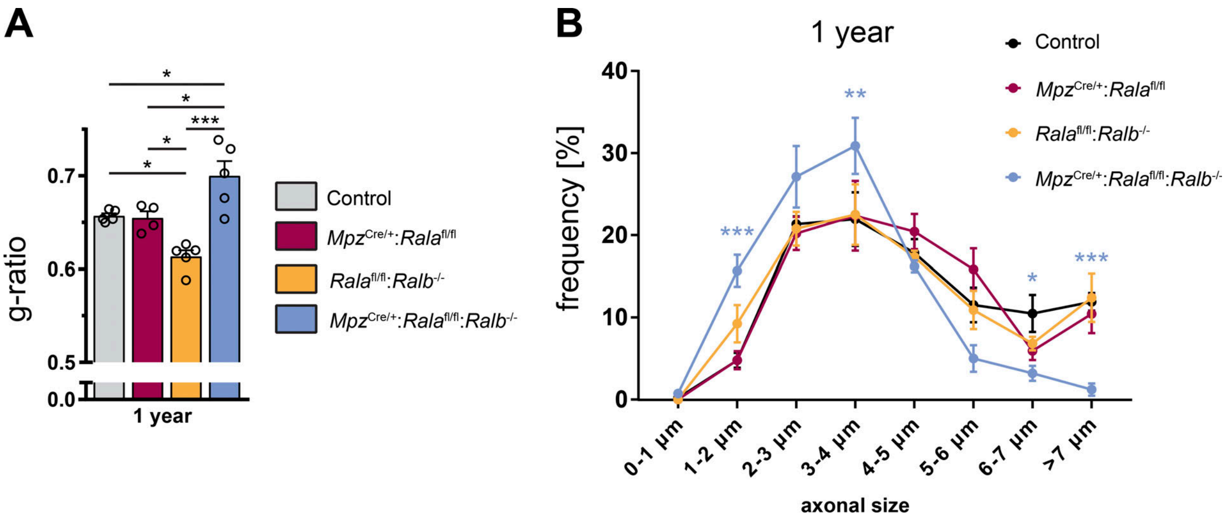

Figure S3. **Alterations in axon-myelin units in 1-yr-old *Mpz*$^{Cre/+}$:*Rala*$^{fl/fl}$:*Ralb*$^{-/-}$ mice. (A)** Average g-ratio measured on 1-yr-old sciatic nerve cross sections (same dataset as shown in Fig. 3 E). *n* = 4 (only *Mpz*$^{Cre/+}$:*Rala*$^{fl/fl}$) or 5 mice per genotype, ≥100 axons per animal analyzed from four randomly selected fields. One-way ANOVA with Tukey's multiple comparisons test. **(B)** Frequency distribution of the diameter of axons as randomly selected for g-ratio measurements at 1 yr of age. *n* = 4 (only *Mpz*$^{Cre/+}$:*Rala*$^{fl/fl}$) or 5 mice per genotype, with ≥100 axons per animal analyzed from four randomly selected fields. Two-way ANOVA with Dunnett's multiple comparisons test. The indicated significance levels in B show the significance of *Mpz*$^{Cre/+}$:*Rala*$^{fl/fl}$:*Ralb*$^{-/-}$ versus controls. All data are shown as mean ± SEM. *, P < 0.05; **, P < 0.01; ***, P < 0.001.
